# The Growth and Aggressive Behavior of Human Osteosarcoma Is Regulated by a CaMKII-Controlled Autocrine VEGF Signaling Mechanism

**DOI:** 10.1371/journal.pone.0121568

**Published:** 2015-04-10

**Authors:** Paul G. Daft, Yang Yang, Dobrawa Napierala, Majd Zayzafoon

**Affiliations:** 1 Department of Pathology, University of Alabama at Birmingham, Birmingham, Alabama, United States of America; 2 Institute of Oral Health Research, Department of Oral and Maxillofacial Surgery, School of Dentistry, University of Alabama at Birmingham, Birmingham, Alabama, United States of America; Second University of Naples, ITALY

## Abstract

Osteosarcoma (OS) is a hyperproliferative malignant tumor that requires a high vascular density to maintain its large volume. Vascular Endothelial Growth Factor (VEGF) plays a crucial role in angiogenesis and acts as a paracrine and autocrine agent affecting both endothelial and tumor cells. The alpha-Ca^2+^/Calmodulin kinase two (α-CaMKII) protein is an important regulator of OS growth. Here, we investigate the role of α-CaMKII-induced VEGF in the growth and tumorigenicity of OS. We show that the pharmacologic and genetic inhibition of α-CaMKII results in decreases in VEGF gene expression (50%) and protein secretion (55%), while α- CaMKII overexpression increases VEGF gene expression (250%) and protein secretion (1,200%). We show that aggressive OS cells (143B) express high levels of VEGF receptor 2 (VEGFR-2) and respond to exogenous VEGF (100nm) by increasing intracellular calcium (30%). This response is ameliorated by the VEGFR inhibitor CBO-P11, suggesting that secreted VEGF results in autocrine stimulated α-CaMKII activation. Furthermore, we show that VEGF and α-CaMKII inhibition decreases the transactivation of the HIF-1α and AP-1 reporter constructs. Additionally, chromatin immunoprecipitation assay shows significantly decreased binding of HIF-1α and AP-1 to their responsive elements in the VEGF promoter. These data suggest that α-CaMKII regulates VEGF transcription by controlling HIF-1α and AP-1 transcriptional activities. Finally, CBO-P11, KN-93 (CaMKII inhibitor) and combination therapy significantly reduced tumor burden *in vivo*. Our results suggest that VEGF-induced OS tumor growth is controlled by CaMKII and dual therapy by CaMKII and VEGF inhibitors could be a promising therapy against this devastating adolescent disease.

## Introduction

Osteosarcomas (OS) are the most frequently diagnosed primary bone tumors in humans [1]. They are hyperproliferative malignant tumors that require high vascular density to maintain their excessively large volume [2]. These tumors commonly occur during the second decade of life, and account for 5% of all pediatric tumors, and 20% of all bone tumors [3]. OS most frequently develop in the highly proliferative metaphyseal region of long bones, commonly coinciding with the adolescent growth spurt [4]. With patient prognosis remaining stagnant since the introduction of multi-agent chemotherapy, it is necessary to identify novel molecular targets to combat this devastating childhood disease.

Ca^2+^/Calmodulin-dependent kinase II (CaMKII) is a ubiquitously expressed multifunctional serine/threonine kinase, crucial for Ca^2+^ signal transduction [5]. It phosphorylates a variety of substrates, which are related to many aspects of cellular function in response to Ca^2+^ signaling [6]. The role of CaMKII in many human cancers has been previously described [7–12]. We were the first to report that the alpha splice variant (α-CaMKII) plays a critical role in determining the aggressive phenotype of OS [13,14]. However, the mechanisms of its action remain unknown.

Excessive tumor growth results in increased demand for oxygen and nutrients which are provided by tumor’s vascular supply [15]. Hence, the ability of tumors to induce new blood-vessel formation has been a major focus of research over the past two decades. It is now known that members of the vascular endothelial growth factor (VEGF) family are some of the major inducers of angiogenesis [16]. VEGF is known to act locally on the tumor microenvironment (paracrine), by binding to VEGF receptors 1 and 2 (VEGFR-1 and VEGFR-2) on endothelial cells leading to their subsequent recruitment, proliferation and migration [17]. The paracrine mechanism of VEGF action dramatically increases the number and size of blood vessels, providing ample blood supply to the tumor. Furthermore, VEGF has been shown to bind to VEGFRs on VEGF-secreting tumor cells (autocrine effect) in a variety of cancers [18–22]. This autocrine response leads to the activation of different signaling pathways, ultimately supporting the growth and proliferation of these tumors. To date, several anti-VEGF drugs, alone or in combination with chemotherapy, have shown promising clinical anti-cancer efficacy in colorectal [23], breast [24], ovarian [25] and glioma [26], validating the potential role of VEGF pathway inhibitors as an emerging therapy for cancer.

Transcriptional regulation of the VEGF promoter is tightly controlled in both normoxic and hypoxic conditions [27]. The VEGF promoter is known to be controlled by many transcription factors, mainly hypoxia inducible factor-1 alpha (HIF-1α) and Activating Protein-1 (AP-1), which together have four consensus binding sequences on the human VEGF promoter [28]. We were the first to report that the knockdown of α-CaMKII in OS cells decreases AP-1 protein levels and the vascularization of intratibially grown tumors in a xenograft mouse model [13, 14]. These findings coupled with published reports describing the role of CaMKII in the expression of hypoxia-inducible genes [29]; suggest that CaMKII-induced growth of OS may be mediated via VEGF.

In this study, we investigated the paracrine and autocrine effects of α-CaMKII-induced VEGF on human OS *in vitro* and *in vivo*. We discovered that the levels of VEGF are augmented with increased aggressiveness of human OS cell lines. Furthermore, we show that α-CaMKII positively regulates the levels of VEGF mRNA and protein in OS cells. We demonstrate that the inhibition of both α-CaMKII and VEGF dramatically decreases the aggressiveness of OS *in vitro* and *in vivo*. Taken together, our findings show that α-CaMKII-induced VEGF is crucial for the growth and aggressiveness of human OS. Furthermore, the combinatorial use of compounds that inhibit both CaMKII and VEGF might be developed into a novel therapeutic approach for the treatment of this devastating childhood tumor.

## Materials and Methods

### Ethics Statement

All mice were used in these studies with the approval of the University of Alabama at Birmingham Institutional Animal Care and Use Committee (APN:140409650).

### Cell Culture and Treatments

Human OS cells (HOS, MG-63, N-methyl-N-nitro-N-nitrosoguanidine (MNNG)/HOS and 143B) and human umbilical vein endothelial cells (HUVEC) were purchased from the American Type Culture Collection (ATCC, Manassas, VA, USA). All cell lines were authenticated by DNA short tandem repeat profiling and experiments were conducted within 6 months of resuscitation. OS cells were maintained in DMEM medium containing 10% FBS (Atlanta Biologicals, Lawrenceville, GA, USA), 100 U/ml penicillin and 100 μg/ml streptomycin (Invitrogen, Carlsbad, CA, USA), while HUVECs were maintained in 200PRF medium (Invitrogen) supplemented with low serum growth supplement (Invitrogen). All cell cultures were maintained at 37°C with 5% CO_2_. 143B cells were serum starved overnight and treated with KN-93 (10 μM) and/or CBO-P11 (1μM) (Millipore) for 24 hours [14].

### Enzyme-Linked Immunosorbent Assay (ELISA)

HOS, MG-63, MNNG/HOS and 143B cells were seeded in 6-well plates at a density of 1 x 10^5^ cells per well and allowed to reach confluency. The supernatants were collected 24 hours later and analyzed for levels of secreted VEGF with a sandwich ELISA (Invitrogen) according to the manufacturer’s instructions. Cells were cultured in serum free media and VEGF protein was normalized to total cellular proteins. The optical density was measured at 450 nm using a Benchmark Plus microplate reader (Bio-Rad, Hercules, USA) [30].

### Tube-like Formation *in vitro* Assay

HUVECs were used at passages 6–8. Each well of a 12-well plate was coated with 300 μl of GELTREX reduced growth factor basement membrane (Invitrogen). The plate was then incubated at 37°C for 30 minutes to allow for GELTREX polymerization. HUVECs (1 x 10^5^/well) were then seeded on the coated plates in a total volume of 500 μl, and incubated with conditioned medium taken from each of the following cell lines cultured in DMEM containing 10% FBS for 48 hours: HOS, GFP-Ctrl, GFP-CaMKIIα, MNNG/HOS, MG-63, 143B, shCtrl or shCaMKIIα. Tube-like formation was documented after 12 hours with photomicrographs taken at 50x magnification. The tube-like length was quantified using ImageJ software (National Institutes of Health, USA) and is shown as percent of total tube-like length [31].

### RNA Extraction and real-time PCR

Total RNA was extracted using the TRIzol method as recommended by the manufacturer (Invitrogen). One μg of RNA was reverse-transcribed using M-MLV reverse transcriptase, and the equivalent of 10 ng was used for SYBR Green real-time quantitative RT-PCR. The expression of β-Actin was used for normalization of gene expression values. The following primers were used for PCR analysis: VEGF, forward 5’-TGCAGATTATGCGGATCAAACC-‘3 and reverse 5’-TGCATTCACATTTGTTGTGCTGTAG-‘3; and Actin, forward 5’- ATTGCCGACAGGATGCAGAA-3’ and reverse 5’-ACATCTGCTGGAAGGTGGACAG-‘3 [14].

### Whole Cell Protein Extraction and Western Blot Analysis

Cells were lysed in 0.5% Nonidet P-40 lysis buffer supplemented with protease and phosphatase inhibitors (Sigma-Aldrich, St. Louis, MO, USA). Following electrophoresis, proteins were transferred to a polyvinylidene difluoride membrane, Immobilon-P (Millipore Co., Milford, MA, USA). Membranes were blocked with Tris-buffered saline-Blotto/Blotto B (Santa Cruz Biotechnology, Santa Cruz, CA, USA) for 1 hour and subsequently incubated overnight with antibodies directed against α-CaMKII, p-α-CaMKII, p-CREB, CREB, p-c-Jun, c- Fos, Lamin B1, HIF-1α, VEGFR-1, VEGFR-2 or β-actin (Santa Cruz Biotechnology and Cell Signaling Technology, Beverly, MA, USA). Signals were detected using a horseradish peroxidase-conjugated secondary antibody and an enhanced chemiluminescence detection kit (ECL; Amersham Biosciences, Pittsburgh, PA, USA) [14]. Band density was measured using ImageJ software and normalized to β-Actin.

### Immunohistochemistry

Tumor containing tibiae of mice treated with vehicle, CBO-P11 and/or KN-93 were collected, formalin fixed, EDTA decalcified, and paraffin embedded. Sectioned bone tissues were then deparaffinized and rehydrated followed by antigen retrieval using 10mM sodium citrate buffer, pH 6. Samples were blocked for 1 hour in 5% goat serum (Vector Laboratories, Burlingame, CA, USA). Antibodies directed against KI-67 (Thermo Fisher Scientific, Waltham, MA, USA) and CD-31 (Abcam, Cambridge, England) were applied to sections and incubated overnight at 4°C. Biotin-conjugated secondary antibodies (2μg/ml) were added, followed by avidin-biotin enzyme reagents. Specimens were incubated in 3,3'-Diaminobenzidine (DAB) peroxidase substrate for 30 seconds. Tissues were counterstained with Gill’s hematoxylin for 10 seconds, dehydrated, cleared and mounted. Rabbit IgG negative controls were processed alongside the examined tissue. Photomicrographs were taken using a Nikon DS-Fi1 digital camera [14].

### Motility Assay

143B Cells were grown to 100% confluency in 6-well plates and scratched with the narrow end of a sterile P200 pipette tip. Medium was changed to remove floating cells and replaced with DMEM medium containing 1% FBS and CBO-P11 (1μM) and/or KN-93 (10μM). Photomicrographs were taken and the scratch width was measured immediately after initial wounding. Cells were then incubated at 37°C with 5% CO_2_. After 12 hours, photomicrographs were taken at 50x magnification and the scratch width was measured. Data were expressed as percentage of the remaining width of the scratch (after 12 hours) when compared to the original width (at 0 hour). Migration analysis was performed using the manual tracking suite in ImageJ [14].

### Invasion Assay

Cells (2.5x10^4^) were plated in media containing 0.1% FBS and CBO-P11 (1μM) and/or KN-93 (10μM) onto the Matrigel coated upper chambers of transwell invasion assay filter inserts (BD Bioscience, East Rutherford, NJ, USA). Medium containing 10% FBS was added into the lower chambers, acting as a chemoattractant. The cells were allowed to invade for 24 hours, after which the cells that invaded the Matrigel were fixed in methanol and stained with crystal violet (Cellgro, Manassas, VA, USA). Representative photomicrographs were taken at 100x magnification. Cells were counted from 5 low-power fields per filter insert [14].

### MTT Assay

Cells were plated at a density of 5x10^3^ cells per well in 96-well plates. After treatment with CBO-P11 (1μM) and/or KN-93 (10μM) for 1, 2, 3, 4 or 5 days, MTT solution [3-(4,5-dimethylthiazol-2-yl)-2,5-diphenyltetrazoliumbromide] (ATCC) was added to the culture medium at a final concentration of 0.5 mmol/L and plates were incubated for 2 hours at 37°C with 5% CO_2_. Detergent solution was then added to solubilize formazan crystals. Finally, the optical density was determined at 570 nm using a Benchmark Plus microplate reader (Bio-Rad, Hercules, USA) [14].

### Trypan Blue Exclusion

To determine the influence of VEGF and CAMKII inhibition on proliferation, 143B cells were plated in DMEM supplemented with 1%FBS at a density of 5x10^3^ cells per well in 96-well plates. Fresh media containing CBO-P11 (1μM) and KN-93 (10μM) was added daily for 5 days. Cells were then detached using trypsin/EDTA, centrifuged and resuspended in PBS. To determine the cell number, a trypan-blue exclusion assay was conducted as previously described [32].

### Measurement of intracellular free Ca^2+^ by Fluo-4 NW

The Fluo-4 NW Calcium Assay Kit (Invitrogen) was used according to manufacturer’s instructions. 143B cells were seeded on poly-D-lysine (PDL)-coated 8-well microscopy chamber cover slides at 1 x 10^4^ cells per well and cultured overnight. Cells were then incubated at 37°C for 30 minutes in the dye loading solution dissolved in assay buffer (Component C, Invitrogen). Following incubation, the cells were washed twice with assay buffer, and equilibrated at room temperature for an additional 30 minutes. [Ca^2+^]_i_ was monitored using the ZEISS LSM 710 laser confocal microscope for 4 min with a 0.1-second interval between measurements and every 40th time point was recorded for analysis. Relative fluorescent intensities at 4 regions-of-interests were measured and graphed as relative fluorescence vs. time [33].

### Gene Silencing by shRNA

143B cells were transduced with lentiviral vectors expressing a control non-functioning scrambled shRNA (shCtrl) or α-CaMKII-targeting shRNA (shCaMKIIα). 143B cells were plated at a density of 2 x 10^4^ cells/cm^2^ in 6-well plates. Cells were incubated in Polybrene (8 mg/mL; Sigma-Aldrich) overnight at 37°C with 5% CO_2_. Two specific α-CaMKII shRNAs or one nonspecific scrambled control shRNA (shCtrl) were cloned into lentiviral transduction vectors (Sigma-Aldrich) and added to the media. The media was changed 24 hours post-transfection, and the transfected cells were cultured with fresh media containing puromycin (5 mg/mL) for selection (Sigma-Aldrich). Once all non-transfected cells died guaranteeing a pure culture, the transfected cells were split into 10 cm plates and maintained stably in culture [14].

### Retrovirus Production and Infection

HOS cells were transduced with a retroviral construct overexpressing GFP-α-CaMKII (GFP-CaMKIIα) or GFP alone (GFP-Ctrl) as a control. Retroviral expression vectors that express either pMSCV-green fluorescent protein (GFP-Ctrl) or pMSCV-GFP-α-CaMKII (GFP-CaMKIIα) were used [14]. The pMSCV-GFP and pMSCV- GFP-α-CaMKII were provided as a gift by Dr. Tobias Meyer at Stanford University Medical Center [34]. Retroviruses were produced by co-transfecting pMSCV vectors with pVSV-G into BOSC23 cells using Lipofectamine (Invitrogen). Twenty-four hours post transfection, the media was replaced, and retroviral supernatant was collected. For infection, 2×10^4^ cells/cm^2^ HOS cells were plated into 6-well plates. The culture media were replaced with 500 μl of retroviral supernatant containing 8 μg/ml Polybrene (Sigma-Aldrich) and cells were incubated for 2 hours at 37°C in 5% CO_2_. Retroviral supernatant was then removed and cells were cultured in regular growth medium [14].

### Chromatin Immunoprecipitation (ChIP) assay

143B OS cells were cultured as described above. Cells were fixed with 1% formaldehyde at room temperature for 10 minutes in order to cross-link DNA protein complex. Nuclei from cross-linked cells were resuspended in Tris-EDTA buffer and sonicated (Fisher Sonic dismembrator, Model 500). The soluble chromatin was resuspended in RIPA buffer (0.1% sodium dodecyl sulfate, 1% Triton X-100, 0.1% sodium deoxycholate, 140 mM NaCl) and immunocleared with 2 μg of salmon sperm DNA/Protein A agarose beads (Upstate Biotechnology) for 1 hr at 4°C. Immunoprecipitation was performed with antibodies directed against HIF-1α, c-Fos and normal rabbit or mouse IgG overnight at 4°C, followed by adding salmon sperm DNA/protein A agarose for 1 hr. Immunoprecipitates were sequentially washed with the following buffers: once with low salt buffer (0.1% SDS, 1% Triton X-100, 2 mM EDTA, 20 mM Tris-HCl (pH 8.1) and 150 mM NaCl), once with high salt buffer (0.1% SDS, 1% Triton X-100, 2 mM EDTA, 20 mM Tris-HCl (pH 8.1) and 500 mM NaCl), once with LiCl buffer (0.25% LiCl, 1% NP-40, 1% Na-Deoxycholate, 1 mM EDTA and 10 mM Tris-HCl (pH 8.1)) and twice with Tris-EDTA buffer. Cross-linking was reversed by heating with 0.2 M NaCl at 65°C overnight. DNA was precipitated with Phenol/Chloroform and the DNA template was amplified by conventional PCR. The following primers were used for PCR analysis: VEGF promoter -1215 to -881 (HRE-containing region), forward 5'-TTGGGCTGATAGAAGCCTTG-3' and reverse 5'- TGGCACCAAGTTTGTGGAGC -3'; VEGF promoter -1814 to -1458 (TRE containing region) forward 5'-GCTCCAGATGGCACATTGTC-3' and reverse 5'- GGAATCCTGGAGTGACCCCT-3'; VEGF promoter -780 to -590 (TRE containing region) forward 5'-GCCGACGGCTTGGGGAGATG-3' and reverse 5'- TCCGGCGGTCACCCCCAAAA-3'. The product was separated by 1.5% agarose gel electrophoresis [35].

### Transient transfections and luciferase reporter assays

143B OS cells were plated at a density of 2 × 10^4^ cells/cm^2^ in 6-well plates. Twenty-four hours after plating, cells were transfected with 1 μg of HRE or TRE luciferase plasmid (Clontech, Palo Alto, CA) using the Amaxa nucleofactor II device (Lonza, Basel, Switzerland) according to the manufacturer's instruction. Transfected cells were then serum starved for 24 hours and treated with CBO-P11 (1μM) and/or KN-93 (10μM) for 24 hours. Cells were lysed and reporter activity was measured using a luciferase assay system (Promega, Madison, WI). Data is expressed as Relative light unit normalized to total protein [33].

### Animal studies and tumor cell inoculation

Six-week-old male Foxn1^nu^ mice (Harlan Laboratories, Indianapolis, IN, USA) were used in these studies, with the approval of the University of Alabama at Birmingham Institutional Animal Care and Use Committee (APN:140409650). 143B cells were prepared from sub-confluent cultures. Cells (1 x 10^6^ cells in 25 μl PBS) were intratibially injected using insulin syringes with 28.5 gauge needles. The knee was flexed, and the needle inserted into the tibia, boring the needle through the epiphysis and epiphyseal growth plate for delivery of the cells into the metaphysis. Tumors were allowed to grow for 7 days. Mice were then implanted with Alzet micro-osmotic pumps (model 1002) (DURECT Corp) that allow for consistent drug delivery for 2 weeks. The reservoir of each pump was filled with 5μg/μl KN-93, 10μg/μl CBO-P11 or vehicle and was set to release 0.25 μl/1 hour (6 μl/24 hours). 143B OS tumor growth was monitored by *in vivo* bioluminescence imaging at 7 and 21 days after cancer cell inoculation by injecting mice with D-luciferin solution (150 mg/kg) 10 minutes before imaging. Images were then acquired and analyzed with an IVIS 100 Imaging System (Xenogen). Regions of interest were identified and plotted as fold difference in tumor size at day 21 compared with day 7. At the end of the study, animals were euthanized by isoflurane inhalation followed by cervical dislocation, hind limbs were excised, formalin fixed, EDTA decalcified, and paraffin embedded. Tissues were sectioned and stained with hematoxylin and eosin (H&E) for histologic evaluation. Photomicrographs were taken using a Nikon DS-Fi1 digital camera [14].

### Statistical Analysis

Statistical analyses were performed using the Microsoft Excel data analysis program for Student’s t-test analysis. Experiments were repeated at least three times, unless otherwise stated. Values were expressed as mean ±SD with results considered significant at p<0.05 [14].

## Results

### VEGF expression and secretion are increased in the highly aggressive OS cell lines

**Fig 1:**
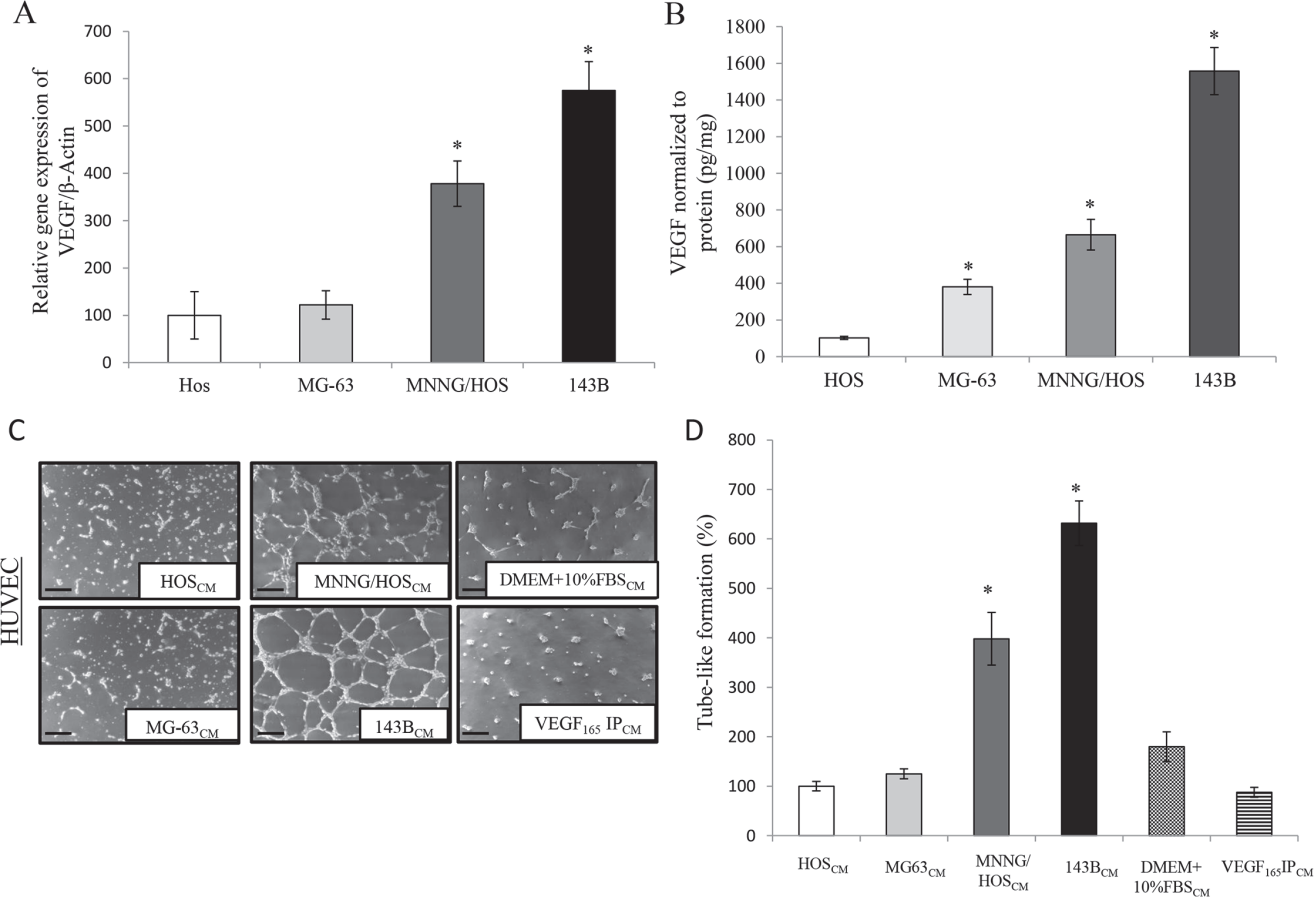
VEGF expression and secretion are increased in the highly aggressive OS cell lines in comparison with non-aggressive OS cell lines. **A.** Real-time PCR was performed using primers specific for VEGF or β-Actin in the human OS cell lines HOS, MG-63, MNNG/HOS and 143B. Values were obtained from three separate experiments each repeated in triplicate and represent the mean ±S.D. *p<0.01. **B.** HOS, MG-63, MNNG/HOS and 143B human OS cells were seeded at 1 x 10^5^ cells per well on a 6-well plate. Twenty-four hours later, conditioned media were examined for the presence of human VEGF by ELISA. Values were obtained from three separate experiments each replicated in triplicate and represent the mean ±S.D. *p<0.01. **C.** HOS, MG-63, MNNG/HOS and 143B human OS cells were seeded at 1 x 10^5^ cells per well on a 6-well plate. Twenty-four hours later, aliquots of supernatant were removed from dishes and added to 12-well plates seeded with 1 x 10^5^ HUVEC cells. Endothelial cell tube-like formation was examined at 12 h. Negative controls of non-conditioned culture media (DMEM + 10% FBS) and conditioned media depleted from VEGF were also included. Representative photomicrographs were taken at 50x magnification from 3 independent experiments, each repeated in triplicate. Scale bar = 50 μm **D.** Capillary tube-like length was quantified using the ImageJ software. Values were obtained from three separate experiments each repeated in triplicate and represent the mean ±S.D. *p<0.01.

To examine the levels of VEGF in human OS, real-time PCR and ELISA were performed using several human OS cell lines (HOS, MG-63, MNNG/HOS and 143B). Here, we show by real-time PCR that the highly aggressive OS cell lines (MNNG/HOS and 143B) express higher levels of VEGF when compared to less aggressive cell lines (MG-63 or HOS). VEGF mRNA levels are 200% higher in the highly aggressive 143B cell line when compared to HOS cells (Fig 1A). To determine VEGF protein levels, OS cells were seeded at a concentration of 1 x 10^5^ cells per well, cultured for 24 hours followed by the collection of conditioned media. Consistent with real-time PCR results, we discovered by ELISA that the levels of secreted VEGF in media collected from 143B cells is 1,500% higher than in media collected from HOS cells (Fig 1B). Finally, the ability of OS-secreted VEGF to induce endothelial tube-like formation was evaluated. HUVECs were cultured on a reduced growth factor basement membrane and treated with conditioned media collected from four different OS cell lines. Here we show that media collected from the highly aggressive OS cell lines (MNNG/HOS or 143B) formed 400–650% more endothelial tube-like networks when compared to conditioned media collected from less aggressive OS cells (HOS or MG-63), (Fig 1C and 1D). To specifically demonstrate that VEGF in the conditioned media is responsible for the tube-like formation we used two additional controls: 1) DMEM fresh media supplemented with 10% FBS; and 2) conditioned media collected from 143B cells after removing VEGF by immunoprecipitation using a VEGF antibody. Both treatments failed to induce tube-like formation suggesting that VEGF secreted from the cultured 143B OS cells is the responsible factor for endothelial tube-like formation. Taken together, this data demonstrate that the highly aggressive human OS cell lines express and secrete higher levels of VEGF that increases endothelial tube-like formation *in vitro*.

### α-CaMKII promotes OS angiogenesis by up-regulating expression and secretion of VEGF

**Fig 2:**
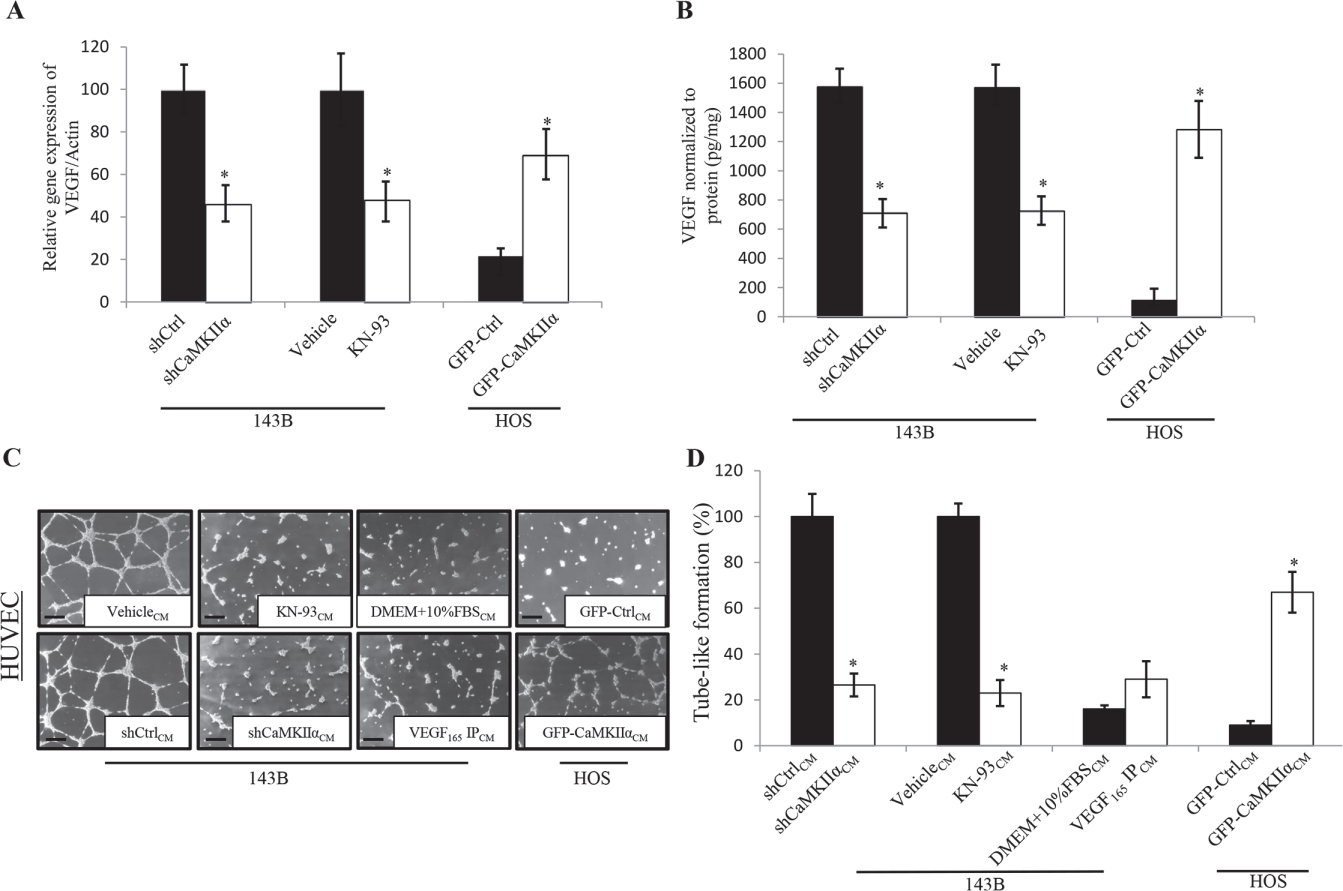
VEGF expression is positively regulated by CaMKII in human OS. 143B cells were transduced with lentiviruses expressing either scrambled (shCtrl) or α-CaMKII- targeting shRNAs (shCaMKIIα) or treated with the CaMKII inhibitor KN-93. HOS cells were transduced with retroviruses expressing either GFP (GFP-Ctrl) or CaMKIIα (GFP- CaMKIIα). **A.** Real-time PCR was performed using primers specific for VEGF or β-Actin. Values were obtained from three separate experiments each repeated in triplicate and represent the mean ±S.D. *p<0.01. **B.** α-CaMKII-inhibited 143B (shCaMKIIα or KN-93) and α-CaMKII overexpressing HOS (GFP-CaMKIIα) cells were seeded at 1 x 10^5^ cells per well of a 6-well plate. Twenty-four hours later, aliquots of supernatant were examined for human VEGF by ELISA. Values were obtained from three separate experiments each repeated in triplicate and represent the mean ±S.D. *p<0.01. **C.** α-CaMKII inhibited 143B (shCaMKIIα or KN-93) and α- CaMKII overexpressing HOS (GFP-CaMKIIα) cells were seeded at 1 x 10^5^ cells per well of a 6- well plate. Twenty-four hours later, aliquots of conditioned media were removed and added to 12-well plates seeded with 1 x 10^5^ HUVEC cells. Endothelial cell tube-like formation was measured at 12 h. Negative controls of non-conditioned culture media (DMEM + 10% FBS) and conditioned media with VEGF immunoprecipitated out (VEGF IP) were also included. Representative photomicrographs were taken at 50x magnification from 3 independent experiments, each repeated in triplicate. Scale bar = 50 μm **D.** Capillary tube-like length was quantified using the ImageJ software. Values were obtained from three separate experiments each repeated in triplicate and represent the mean ±S.D. *p<0.01.

We have previously shown that α-CaMKII levels positively correlate with the aggressive phenotype of human OS cell lines *in vitro* and with the ability of OS tumors to form blood vessels *in vivo* [14]. In order to determine whether α-CaMKII directly regulates OS tumor microvascular density through VEGF, we knocked down α-CaMKII in the highly aggressive human OS cell line 143B, and overexpressed α-CaMKII in the non-aggressive OS cell line (HOS). Furthermore, we inhibited CaMKII using a pharmacologic antagonist KN-93 (10μM). We previously reported the efficiency of shCaMKIIα and GFP-CaMKIIα in modifying the levels of CaMKIIα in OS cells [14]. Here we show, that α- CaMKII inhibition and knockdown decrease VEGF gene expression (50%), while α-CaMKII overexpression in HOS cells increases VEGF gene expression (250%) when compared to controls (Fig 2A). Additionally, we show that α-CaMKII knockdown or inhibition in 143B cells result in a 55% or 52% decrease in VEGF protein secretion, respectively, when compared to controls, while GFP-CaMKIIα HOS cells secrete 1,200% more VEGF than GFP-Ctrl (Fig 2B). Furthermore, conditioned media collected from 143B cells with α-CaMKII depleted by shRNA or inhibited by KN-93 demonstrate a decreased ability to induce endothelial tube-like network formation (~80%) when compared to controls, while conditioned media collected from HOS cells overexpressing α-CaMKII increases endothelial tube-like network formation (~700%) when compared to control (Fig 2C and 2D). DMEM media supplemented with 10% FBS as well as conditioned media collected from 143B cells after removing VEGF by immunoprecipitation using a VEGF antibody were used as controls to verify that VEGF in the media is responsible for the tube-like formation. The conditioned media after either treatment failed to induce tube-like formation suggesting that VEGF secreted from the cultured 143B OS cells is the responsible factor for endothelial tube-like formation (Fig 2C and 2D).

### OS cell-secreted VEGF has an autocrine effect on tumor cells

**Fig 3:**
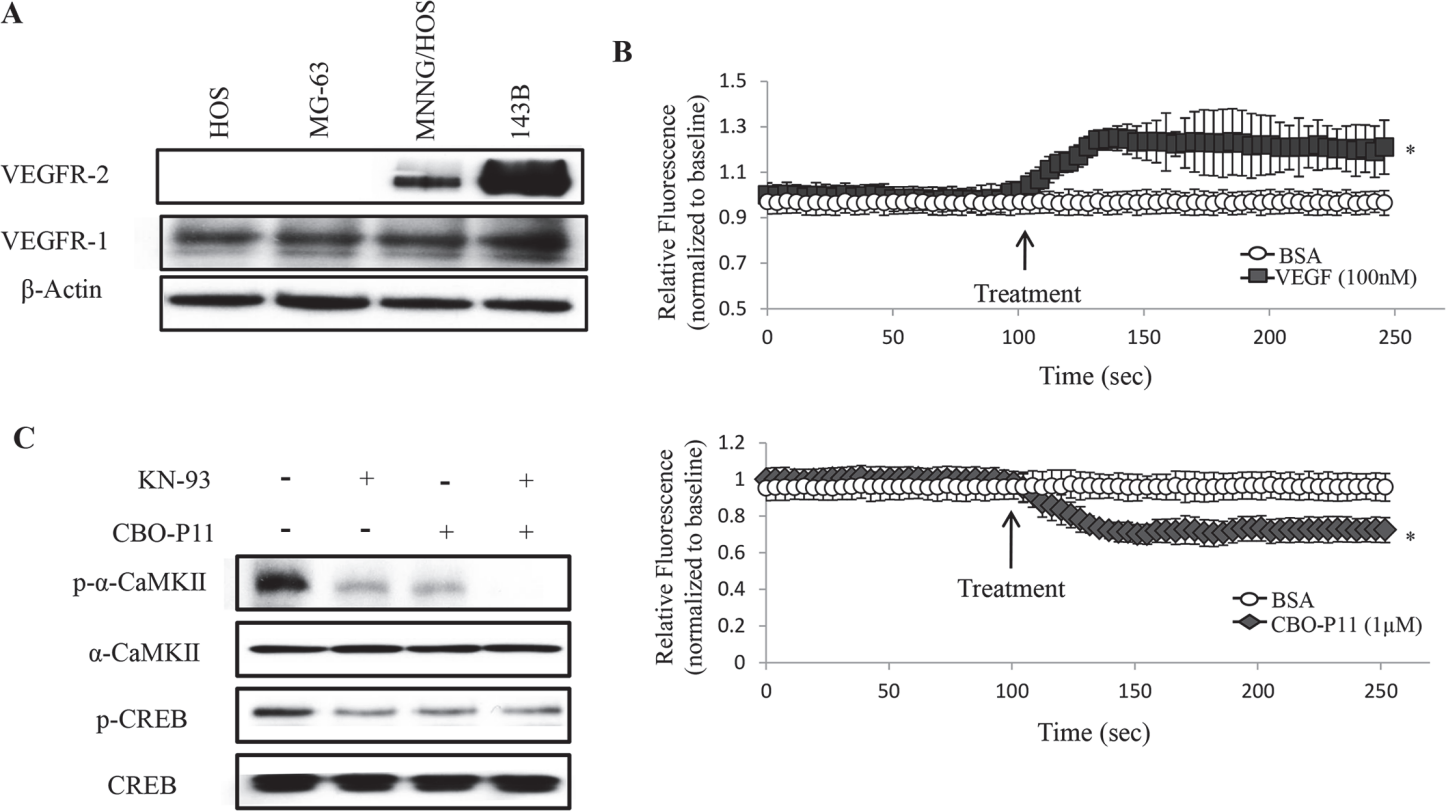
VEGF acts on 143B human OS cells in an autocrine manner. **A.** VEGF receptors were identified by western blot. Immunoblots were developed using specific antibodies directed against VEGFR-2, VEGFR-1 or β-Actin. The autoradiograph is representative of three experiments. **B.** Detection of [Ca^2+^]_i_. 143B cells were incubated with 5μM of Fluo-4-AM and then treated with 100nM VEGF (upper panel) or 1μM CBO-P11 (lower panel). BSA was used as control. Relative fluorescence intensities at four regions-of-interests were measured, with the average fluorescent intensity plotted versus time. Values were obtained from three separate experiments each repeated in triplicate and represent the mean ±S.D. *p<0.01. **C.** Activation of the CaMKII signaling. 143B Cells were treated with 10 μM KN-93 and/or 1 μM CBO-P11 for 24 hours. Immunoblots were developed using specific antibodies directed against p-α-CaMKII, α-CaMKII, p-CREB or CREB. The autoradiographs are representative of three independent experiments.

The paracrine effect of tumor-secreted VEGF on its surrounding endothelial cells is well established [36]. Interestingly, evidence continues to emerge suggesting an additional autocrine effect of VEGF on tumor, which may contribute to a more severe tumor pathogenesis [37]. An increasing number of primary tumors have been shown to express functional VEGF receptors and respond directly to increases in extracellular VEGF by increasing free intracellular Ca^2+^ [Ca^2+^]_i_ [38]. In order to examine if OS cells are capable of responding to extracellular VEGF, we first examined the levels of VEGFRs in OS cells. Western blot analyses were performed using antibodies directed against VEGFR-1, VEGFR-2 or β-Actin. Here we show that VEGFR-1 is highly expressed in all four of the examined human OS cell lines while VEGFR-2 is only expressed in the more aggressive cell lines (MNNG/HOS and 143B), suggesting that aggressive OS cells are potentially more capable of responding to extracellular VEGF (Fig 3A). We then examined the effects of exogenous VEGF (100nM) on [Ca^2+^]_i_ in 143B cells. Upon treatment with 100nM VEGF, a 30% increase in [Ca^2+^]_i_ was observed when compared to cells treated with BSA (Fig 3B, upper panel), while VEGFR inhibition by 1μM CBO-P11 (competitive VEGF receptor inhibitor) results in a 25% decrease in [Ca^2+^]_i_ when compared to control (Fig 3B, lower panel). Furthermore, we show by western blot analyses that VEGFR inhibition by CBO-P11 (1μM for 24 hours) decreases the activation of α-CaMKII and its downstream signaling target, CREB in 143B OS cells (Fig 3C). This data suggests the presence of a positive signaling feedback loop in OS cells, where extracellular VEGF increases the activation of α-CaMKII and its downstream targets, which ultimately leads to further increases in VEGF expression.

### CaMKII and VEGFR inhibition results in a decrease of OS proliferation, motility and invasion

**Fig 4:**
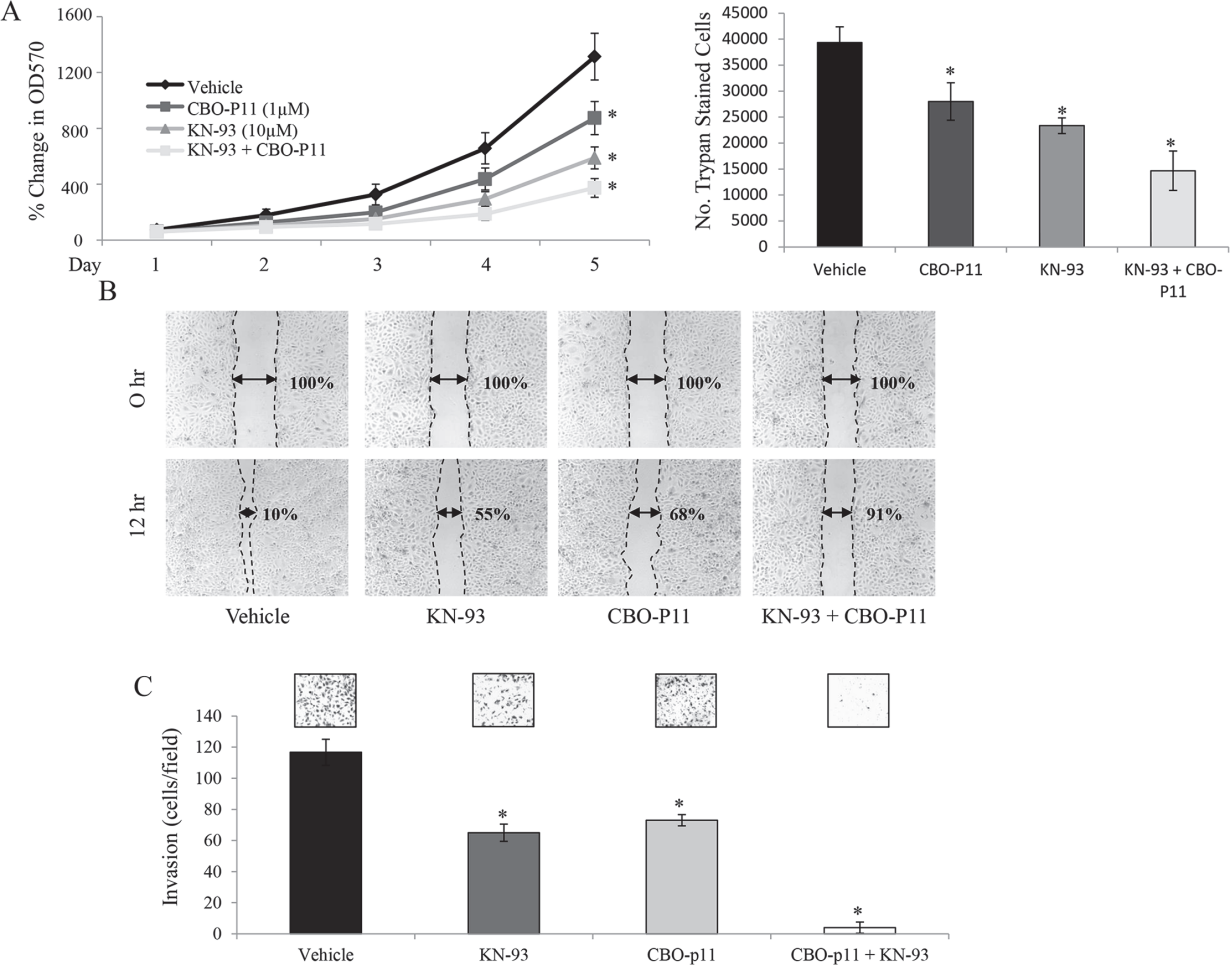
Pharmacologic inhibition of CaMKII and VEGF decreases tumorigenicity of 143B OS cells *in vitro*. **A.** MTT assay (left panel) was performed at days 1, 2, 3, 4 and 5 to determine the number of viable cells. 1 x 10^3^ 143B cells were seeded in a 96-well plate. At 24 hours after seeding, cells were treated with 10μM KN-93 and/or 1μM CBO-P11. Values were obtained from three separate experiments, each repeated in triplicate and represent the mean ± S.D. *p<0.01. Trypan blue exclusion assay (right panel) was performed at day 5 to determine the live cell number. At 24 hours after seeding, cells were treated with 10μM KN-93 and/or 1μM CBO-P11 every 24 hours. Values were obtained from three separate experiments, each repeated in triplicate and represent the mean ± S.D. *p<0.01. **B.** Scratch/wound healing assay was performed on cells cultured for 12 hours. The width between the scratched areas at 0 hour was set to 100%. Representative photomicrographs were taken at 50x magnification from 3 independent experiments, each repeated in triplicate. Values represent the mean ± S.D. *p<0.01. **C**. Transwell invasion assay allowing cells to invade for 24 hours. Representative photomicrographs were taken at 100x magnifications from 3 independent experiments, each repeated in duplicate. Values represent the mean ± S.D. *p<0.01.

The cell number, viability, motility and invasion of 143B OS cells in response to VEGFR and/or CaMKII inhibition were then examined. Using a 5 day MTT assay, we show that the inhibition of both VEGFRs by CBO-P11 and CaMKII by KN-93 (10μM for 24 hours) decreases the viability of 143B cells. On day five of the MTT assay, the cell viability of 143B cells when compared to vehicle treated controls, was significantly lower after the inhibition of CaMKII (59%), VEGFRs (33%), or both (75%) (Fig 4A, left panel). Furthermore, we show that the inhibition of both VEGFRs by CBO-P11 and CaMKII by KN-93 (10μM for 24 hours) decreases 143B cell number when compared to control. On day five of the trypan blue exclusion assay, the 143B cell number was significantly less after the inhibition of CaMKII (38%), VEGFRs (30%), or both (63%) (Fig 4A, right panel). We then examined the motility of 143B OS cells in response to treatment with CBO-P11 and/or KN-93. Motility studies were performed in 1% FBS-supplemented medium in order to suppress cell proliferation and allow us to identify OS cell motility independent of proliferation. A scratch was made at hour 0, and the migration of 143B OS cells into the cell free area was quantitated after 12 hours. Here we show that when VEGFRs or CaMKII is inhibited, 143B cells migrate 64% and 50% less, respectively, when compared to vehicle control, while a combination of both inhibitors decreases migration by 90% (Fig 4B). Finally, invasiveness of 143B OS cells was evaluated using a 24-hour trans-well invasion assay. Here we show that inhibition of CaMKII or VEGFRs decreases invasion by 48% and 44%, respectively, when compared to vehicle treated-control, while combined inhibition decreases invasion by 97% (Fig 4C). These results demonstrate that the inhibition of CaMKII and/or VEGFRs in human 143B OS cells leads to dramatic decreases in cell viability, cell number, motility and invasion.

**Fig 5:**
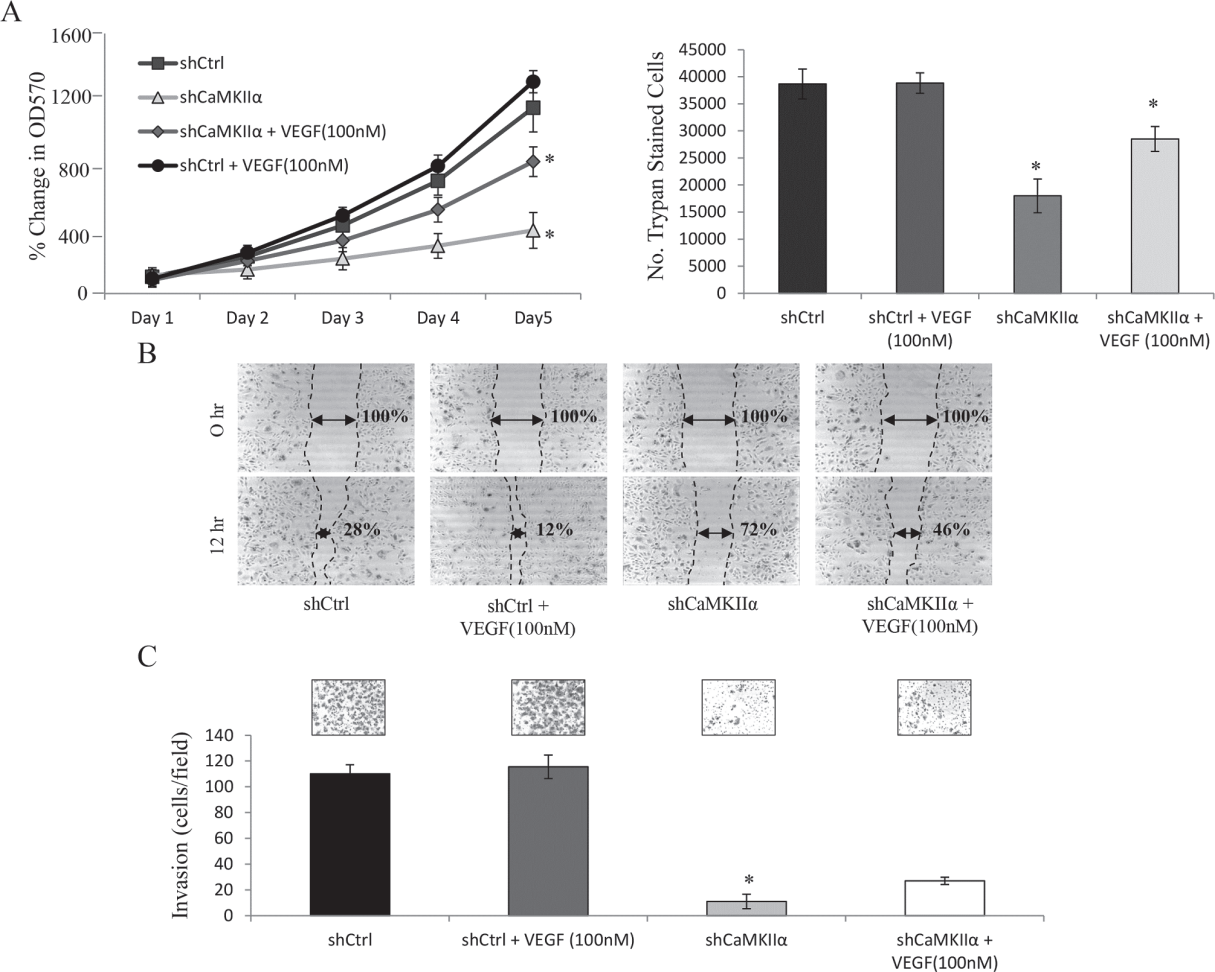
Exogenous VEGF partially rescues normal cell phenotype in α-CaMKII silenced 143B OS cells. 143B cells were transduced with lentiviruses expressing either scrambled (shCtrl) or α-CaMKII- targeting shRNAs (shCaMKIIα) **A.** MTT assay (left panel) was performed at days 1, 2, 3, 4 and 5 to determine the number of viable cells. 5x10^3^ 143B cells were seeded in a 96-well plate. At 24 hours after seeding, cells were treated with 100nM VEGF. Values were obtained from three separate experiments, each repeated in triplicate and represent the mean ± S.D. *p<0.01. Trypan blue exclusion assay (right panel) was performed at day 5 to determine the live cell number. At 24 hours after seeding, cells were treated with 100nM VEGF every 24 hours. Values were obtained from three separate experiments, each repeated in triplicate and represent the mean ± S.D. *p<0.01. **B.** Scratch/wound healing assay was performed on cells cultured for 12 hours. The width between the scratched areas at hour 0 was set to 100%. Representative photomicrographs were taken at 50x magnification from 3 independent experiments, each repeated in triplicate. Values represent the mean ± S.D. *p<0.01. **C**. Trans-well invasion assay allowing cells to invade for 24 hours. Representative photomicrographs were taken at 100x magnifications from 3 independent experiments, each repeated in duplicate. Values represent the mean ± S.D. *p<0.01.

In order to examine whether α-CaMKII-induced VEGF is specifically responsible for the changes in OS tumorigenicity, we depleted α-CaMKII by shRNA and examined the effects of rescuing the tumorigenic phenotype of 143B OS cells by treating with 100nM VEGF. Using an MTT assay, we show that the knockdown of α-CaMKII by shRNA decreases the cell viability of 143B OS cells by 72% and treatment with VEGF restored 66% of this inhibition (Fig 5A, left panel). Using a trypan blue exclusion assay, we show that α-CaMKII knockdown decreases 143B cell number by 53% and treatment with VEGF restored 60% of this inhibition (Fig 5A, right panel). Furthermore, we show that α-CaMKII knockdown decreases 143B OS cell migration by 61% and treatment with VEGF restored 59% of this inhibition (Fig 5B). Finally, we show by an invasion assay that α-CaMKII knockdown decreases the invasion of 143B OS cells by 85% and treatment with VEGF restored 25% of this inhibition (Fig 5C).

### CaMKII and VEGF positively regulate the levels of HIF1-α and AP-1 in OS cells

**Fig 6:**
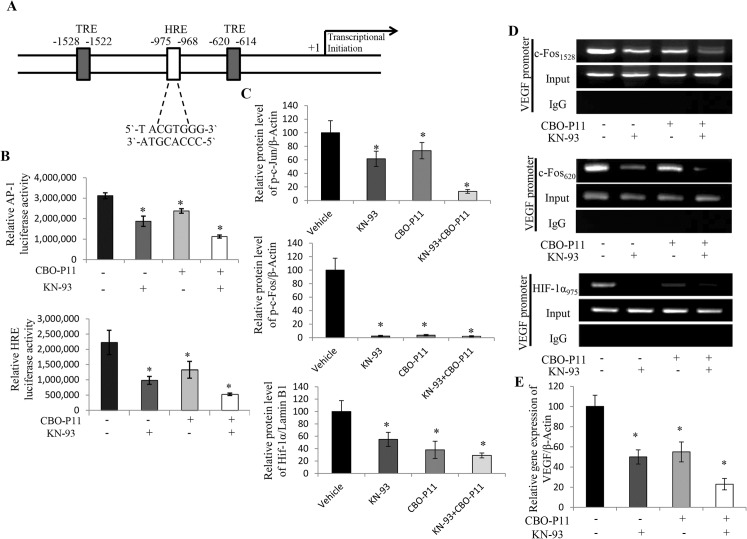
CaMKII controls VEGF gene expression by regulating TRE and HRE. **A.** Schematic illustration of the VEGF promoter showing the binding sites for the AP-1 and HIF-1α transcription factors **B.** Reporter activation assay. Cells were transfected with TRE and HRE luciferase constructs and treated with 10 μM KN-93 and/or 1 μM CBO-P11 for 24 hours and harvested 24 hours later for luciferase activity measurement. Data are expressed relative to total protein, and values represent the mean ± SD of 3 separate experiments each repeated in triplicate; *p≤ 0.01. **C.** Quantification of western blot analyses. 143B cells were treated with 10 μM KN-93 and/or 1 μM CBO-P11 for 24 hours. Immunoblots were developed using specific antibodies directed against p-c-Jun, c-Fos, HIF-1α, Lamin B1 or β-Actin. Band density was measured using imageJ software and normalized to β-Actin. **D.** ChIP assay. DNA-protein complexes were immunoprecipitated with antibodies against AP-1 (c-Fos), HIF-1α and normal rabbit IgG as a control. Immunoprecipitated DNA fragments were amplified by PCR using primers encompassing the TRE and HRE binding sites on the VEGF promoter. Three independent experiments were performed. **E.** Quantitative analyses of the endogenous VEGF Expression. Real-time PCR was performed using primers specific for VEGF or β-Actin in human OS cell lines treated with 10 μM KN-93 and/or 1μM CBO-P11 for 24 hours. Values were obtained from three separate experiments each replicated in triplicate and represent the mean ±S.D. *p<0.01.

The transcriptional regulation of VEGF is a complex and highly controlled process. The VEGF promoter is known to be regulated by HIF-1 and AP-1 transcription factors and their response elements (HRE and TRE, respectively) (Fig 6A) [39]. In order to examine the role of CaMKII and/or VEGF in the regulation of HIF-1α and/or AP-1, we transfected 143B OS cells with 1μg HRE or TRE luciferase reporter plasmids for 24 hours. Cells were then treated with CBO-P11 and/or KN-93, for another 24 hours. At the end of the study, cells were lysed and reporter activity was measured. Here we show that the inhibition of CaMKII by KN-93 or VEGFRs by CBO-P11 decreases AP-1 trans-activation by 33% and 41%, respectively, while dual inhibition results in a 66% decreases in AP-1 transactivation when compared to control. Furthermore, the inhibition of CaMKII or VEGFRs decreases HRE transactivation by 66% and 45%, respectively, while inhibiting both results in 80% decrease in HRE transactivation when compared to control (Fig 6B). Western blot analyses of 143B total protein extracts after treating cells with KN-93 and/or CBO-P11 for 24 hours show that the inhibition of CaMKII and VEGF signaling significantly decreases the levels of Jun (80%), c-Fos (97%) and HIF-1α (70%) proteins (Fig 6C). In order to investigate whether the decreases in the reporters’ activity are due to decreased occupancy of AP-1 and HIF-1 on their DNA response elements in the VEGF promoter, we performed a chromatin immunoprecipitation (ChIP) assay. Cells treated with KN-93 and/or CBO-P11 for 24 hours were fixed in formaldehyde and sonicated. We found that the inhibition of CaMKII and/or VEGFRs decreases AP-1 (TRE1528 and TRE620) and HIF-1α (HRE975) binding on the VEGF promoter of 143B OS cells (Fig 6D). Real-time PCR was performed to confirm that decreases in HIF-1α and AP-1 binding to the VEGF promoter causes decreases in VEGF gene expression. Here we show significant decreases in VEGF gene expression in 143B OS cells after the inhibition of CaMKII (50%), VEGFRs (45%) or both (80%) (Fig 6E). Taken together, these data suggest that CaMKII and VEGF are regulating VEGF gene expression by AP-1 and HIF-1α transcription factors.

### Inhibition of VEGF and/or CaMKII results in decreased OS tumor growth in animal model

**Fig 7:**
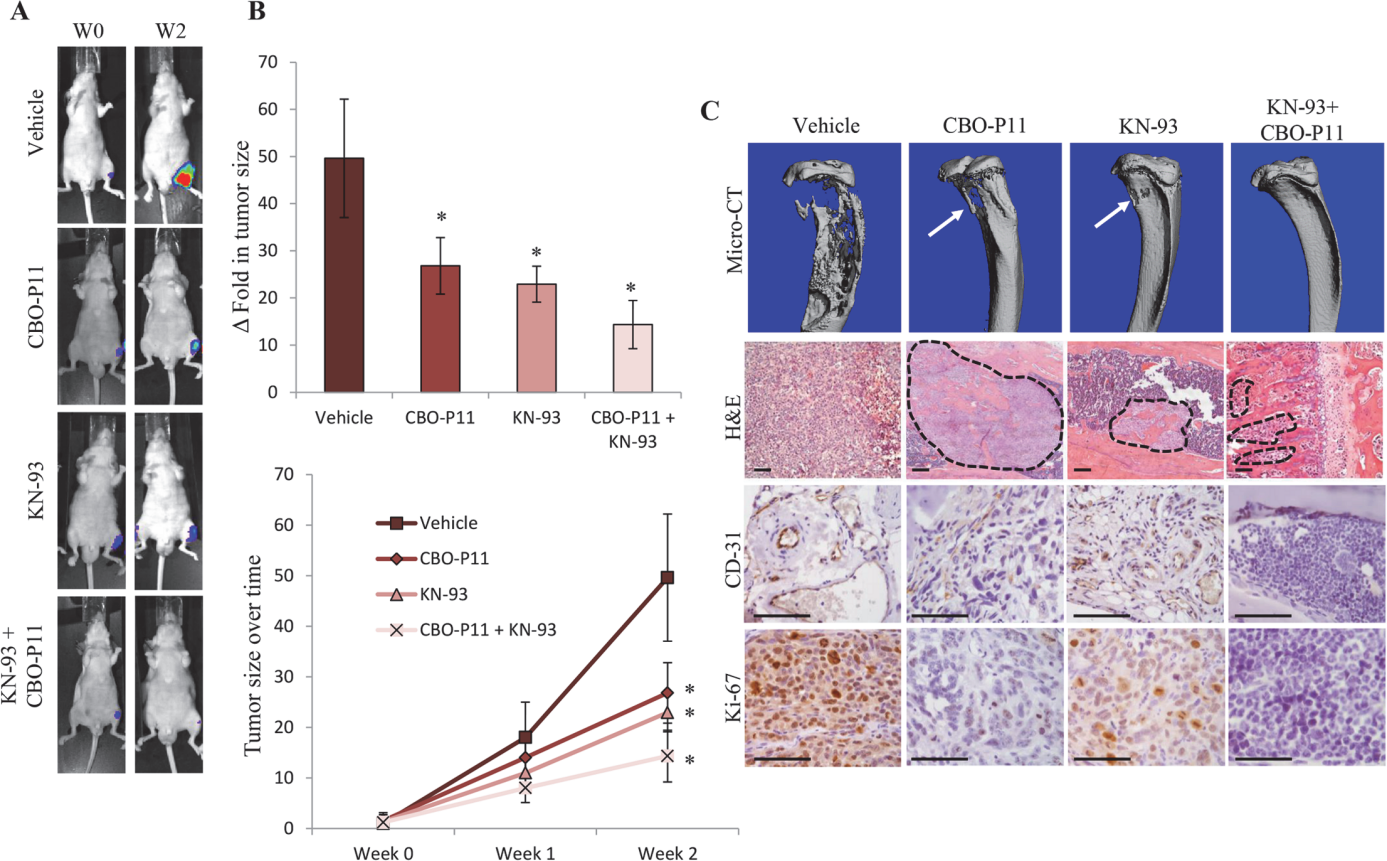
Inhibition of VEGF and CaMKII in OS cells dramatically decreases tumor growth *in vivo*. 143B cells were transduced with lentiviruses expressing firefly luciferase, injected intratibially and allowed to grow for 1 week. ALZET micro-osmotic pumps were subcutaneously implanted into mice delivering saline (vehicle), CBO-P11 (2 mg/kg/day), KN-93 (1 mg/kg/day), or both CBO-P11 and KN-93. **A.** Luciferase imaging was performed before treatment (W0) and 2 weeks after treatment (W2) (n = 8). **B.** Tumor size was measured based on the luminescence intensity and graphed. Values were obtained from 8 mice from each group and represent the mean ± SD. *P < 0.01. **C.** Mice were euthanized and tibiae were collected. Tibiae were scanned by μ-CT, with arrows indicating areas of bone erosion. Hematoxylin and eosin staining was performed on paraffin embedded tumors. Broken lines indicate the boundary of the tumors and separating it from normal bone microenvironment. Images were taken at 40x magnification. IHC staining using specific antibodies directed against CD31 and Ki-67 (brown) were performed. Images were taken at 200x magnification and are representative of 8 different mice. Scale bar = 50 μm.

We next examined whether VEGFR and/or CaMKII inhibition affects the growth of OS cells *in vivo*. We intratibially injected 143B OS cells into 6-week old male athymic (nude) mice. Before injection, these cells were transduced with lentivirus encoding firefly luciferase allowing for *in vivo* monitoring of tumor growth. Tumors were allowed to grow and establish in the bones of the animals for one week (W0). Mice were then randomly divided into four groups receiving saline as control, CBO-P11 (2 mg/kg/day), KN-93 (1 mg/kg/day), or both CBO-P11 and KN-93 for 2 weeks (W2). Subcutaneously implanted ALZET micro-osmotic pumps were used to deliver treatments. Here we show by luminescence imaging that the inhibition of VEGFRs by CBO-P11 or CaMKII by KN-93 reduces tumor burden by 44% and 52%, respectively, while the combined treatment with both KN-93 and CBO-P11 resulted in even greater reductions in tumor size (74%) when compared to that of the saline treated control group (Fig 7A and 7B). At the end of the study (after 2 weeks of treatment), mice were euthanized and tibiae were collected. Tibiae were scanned by μ-CT and later processed for histology and IHC staining. μ-CT analyses confirmed that KN-93 and CBO-P11 treatment dramatically reduced bone destruction caused by the growth of intratibial OS tumors (Fig 7C, Micro-CT panel). H&E staining confirms smaller tumors with decreased bone destruction in CBO-P11 and/or KN-93 treated mice when compared to control (Fig 7C, 7H and 7E panel). Finally, IHC staining was performed using antibodies directed against CD31, an endothelial cell specific protein, and Ki-67, a protein marker for cell proliferation (Fig 7C, lower panels). Here we show CaMKII and VEGFR inhibition dramatically decreases tumor microvasculature and tumor cell proliferation when compared to saline treated controls.

Taken together, our data demonstrate that a-CaMKII-induced VEGF plays a crucial role in the growth and tumorigenicity of OS cells *in vitro* and *in vivo*.

## Discussion

Tumor growth, progression and metastasis are dependent on new blood vessel formation (neo- vascularization). VEGF is a potent angiogenic factor secreted by a variety of tumor cells, and is ubiquitously expressed at sites of angiogenesis [40,41]. It has previously been shown that VEGF expression in OS is predictive of pulmonary metastasis in patients who underwent aggressive therapy [2,42]. Although several studies have demonstrated poor prognosis in patients with high VEGF levels, there have been few studies trying to understand the molecular mechanisms responsible for clinically aggressive behavior in OS. Here we show that the highly metastatic 143B OS cell line has increased VEGF expression and protein secretion when compared to parental HOS cells. Interestingly, it has recently been shown that decreases in HIF-1 by CaMKII inhibition significantly decreases VEGF expression in human macrophages [43]. Our results show a similar effect in 143B cells, where by inhibiting CaMKII genetically or pharmacologically we observe a ~50% decrease in VEGF protein secretion. The dependence of the VEGF levels on the CaMKII levels is confirmed by the overexpression approach. When CaMKII is overexpressed in HOS cells a ~1,000% increase in VEGF protein secretion was observed. These results suggest that CaMKII regulates VEGF in OS, which is consistent with the effect of CaMKII on VEGF previously described in human macrophages [43].

Though VEGF signaling has mainly been described in endothelial cells, there is increasing evidence that VEGF autocrine signaling may play a prominent role in highly metastatic cancer cell lines [44,45]. In this article, we report that the highly metastatic 143B OS cells express VEGFR-2, thereby establishing an autocrine signaling mechanism associated with aggressive phenotypes. 143B cells express higher levels of VEGFR-2 than parental HOS cells and are sensitive to exogenous VEGF stimulation. This autocrine signaling mechanism results in increases in [Ca^2+^]_i_ and leads to increased proliferation, invasion and migration. These results are consistent with previous studies that have shown increases in [Ca^2+^]_i_ and subsequent CaMKII activation in vascular smooth muscle cells and hippocampal neurons upon treatment with exogenous VEGF [46,47]. The inhibition of VEGF-stimulated increases in [Ca^2+^]_i_ and its downstream target, CaMKII was successfully achieved by using VEGFR-2 inhibitor, CBO-P11. These results demonstrate a novel positive feedback loop, where CaMKII-induced VEGF results in increases in [Ca^2+^]_i_ and CaMKII activation.

It was previously reported that Ca^2+^ ionophores result in increased VEGF transcription in human lung carcinoma cells [28]. The human VEGF promoter contains three TRE and one HRE consensus binding regions, which might help explain the molecular mechanisms by which CaMKII is transcriptionally regulating VEGF [48]. AP-1 is a protein complex that binds to the TRE consensus regions, and has been shown to be tightly regulated by CaMKII in human osteoblasts [49]. We discovered that the inhibition of both CaMKII and VEGFR results in decreased HIF-1α and AP-1 binding to the VEGF promoter, which is likely responsible for the decreases observed in VEGF transcription. Furthermore, others have shown that c-fos, in combination with other members of the AP-1 family of transcription factors, regulate the expression of several matrix metalloproteinases, such as MMP-1, -9 and -13 [50]. These changes in MMP expression could be responsible for the decreased invasion observed in response to CaMKII and VEGFR inhibition. Taken together, our results demonstrate the unique role of CaMKII-induced VEGF transcription in the *in vitro* tumorigenicity of OS cells.

Furthermore, we show that the inhibition of CaMKII and VEGF decreases OS tumor growth *in vivo*. The therapeutic benefit of anti-VEGF treatment on solid tumors is well documented. Initially, the addition of Bevacizumab, a monoclonal antibody for VEGF, to standard chemotherapy produced significant clinical benefit in patients with previously untreated and pretreated metastatic colorectal cancer, advanced non-small cell lung cancer, and metastatic breast cancer [51]. Similarly, our data show that the inhibition of CaMKII and VEGF produces not only smaller tumors but also decreases tumor vasculature as demonstrated by a decrease in the number of positively stained CD31 blood vessels. Furthermore, decreases in the osteolytic properties of OS are observed by micro-CT. These findings suggest that combinational treatment with CaMKII and VEGF inhibitors are beneficial for decreasing OS tumor growth and angiogenesis that ultimately result in a dramatic decrease in tumor size.

The current therapy for OS is centered around the combination of chemotherapy and surgery in order to surgically remove as much as possible of the tumor mass and kill the remaining tumor. Our results show that the combined use of CaMKII and VEGFR inhibitors can be used as either pre- or post-surgical intervention. Our data show that the combined treatment will block the ability of the OS tumor to grow and proliferate which will make its resection easier. This approach also stops the tumor’s ability to develop new blood vessels which will ultimately starve the tumor and further decrease tumor size. However, a potential complication of using the combined therapy could arise in response to using antiangiogenic therapy. The decrease in vasculature development within the tumor could negatively affect the ability of cytotoxic drugs to reach their intended targets. Although our preliminary data demonstrate that this is not the case, further studies need to be performed to evaluate this potential risk.

Our findings provide novel insights into the molecular basis of the emerging interplay between CaMKII and tumor angiogenesis, and the novel VEGF/VEGFR-2-mediated autocrine signaling loop. These findings help advance our understanding of the molecular mechanisms associated with poor prognosis in VEGF positive human OS patients, and provide valuable clues for future therapeutic strategies, as OS remains a major therapeutic challenge.
